# Acetic Acid Treatment Enhances Drought Avoidance in Cassava (*Manihot esculenta* Crantz)

**DOI:** 10.3389/fpls.2019.00521

**Published:** 2019-04-24

**Authors:** Yoshinori Utsumi, Chikako Utsumi, Maho Tanaka, Chien Van Ha, Satoshi Takahashi, Akihiro Matsui, Tomoko M. Matsunaga, Sachihiro Matsunaga, Yuri Kanno, Mitsunori Seo, Yoshie Okamoto, Erika Moriya, Motoaki Seki

**Affiliations:** ^1^RIKEN Center for Sustainable Resource Science, Yokohama, Japan; ^2^Core Research for Evolutional Science and Technology, Japan Science and Technology, Kawaguchi, Japan; ^3^RIKEN Cluster for Pioneering Research, Wako, Japan; ^4^Research Institute for Science and Technology, Tokyo University of Science, Noda, Japan; ^5^Department of Applied Biological Science, Graduate School of Science and Technology, Tokyo University of Science, Noda, Japan; ^6^Dormancy and Adaptation Research Unit, RIKEN Center for Sustainable Resource Science, Yokohama, Japan; ^7^Kihara Institute for Biological Research, Yokohama City University, Yokohama, Japan

**Keywords:** ABA, acetic acid, cassava, drought avoidance, drought response

## Abstract

The external application of acetic acid has recently been reported to enhance survival of drought in plants such as *Arabidopsis*, rapeseed, maize, rice, and wheat, but the effects of acetic acid application on increased drought tolerance in woody plants such as a tropical crop “cassava” remain elusive. A molecular understanding of acetic acid-induced drought avoidance in cassava will contribute to the development of technology that can be used to enhance drought tolerance, without resorting to transgenic technology or advancements in cassava cultivation. In the present study, morphological, physiological, and molecular responses to drought were analyzed in cassava after treatment with acetic acid. Results indicated that the acetic acid-treated cassava plants had a higher level of drought avoidance than water-treated, control plants. Specifically, higher leaf relative water content, and chlorophyll and carotenoid levels were observed as soils dried out during the drought treatment. Leaf temperatures in acetic acid-treated cassava plants were higher relative to leaves on plants pretreated with water and an increase of ABA content was observed in leaves of acetic acid-treated plants, suggesting that stomatal conductance and the transpiration rate in leaves of acetic acid-treated plants decreased to maintain relative water contents and to avoid drought. Transcriptome analysis revealed that acetic acid treatment increased the expression of ABA signaling-related genes, such as *OPEN STOMATA 1 (OST1)* and *protein phosphatase 2C*; as well as the drought response and tolerance-related genes, such as the *outer membrane tryptophan-rich sensory protein (TSPO)*, and the *heat shock proteins*. Collectively, the external application of acetic acid enhances drought avoidance in cassava through the upregulation of ABA signaling pathway genes and several stress responses- and tolerance-related genes. These data support the idea that adjustments of the acetic acid application to plants is useful to enhance drought tolerance, to minimize the growth inhibition in the agricultural field.

## Introduction

Abiotic stresses are one of the crucial constraints to food security and crop production. Among them, heat and drought are the two most important stresses with a negative impact on growth and productivity of crops (Fahad et al., 2017). Due to increasing demands for greater food production, it is important to develop desirable and strategic crops, such as cassava, to increase food productivity (Kamanga et al., 2018; Varshney et al., 2018). Drought tolerance is an important research subject pertaining to cassava, as climate change has raised concerns about global drought problems and also places significant demands on breeding programs (Aina et al., 2007; De Oliveira et al., 2017).

Cassava is a tropical crop that serves as an important source of food and industrial materials. It is a vital economic resource for poor farmers in marginal areas where its production is constrained by drought. Although cassava is one of the most drought-tolerant crops, the underlying mechanism for its ability to survive under drought has been investigated recently. For example, physiological association among cassava varieties to drought has been evaluated using plants on field condition and tissue cultures in several countries (Chemonges et al., 2013; Oliveira et al., 2015; Jolayemi and Opabode, 2018). The drought-responsive genes and proteins have been identified in order to understand the physiological mechanisms of drought survival (Utsumi et al., 2012; Turyagyenda et al., 2013; Zhao et al., 2015; Fu et al., 2016). Cassava plants can activate a combination of drought avoidance and tolerance mechanisms that help to maintain optimum growth, development, and metabolism (El-Sharkawy, 2007). Increasing the growth efficiency and survivability of cassava plants subjected to drought, could help the plant survive in marginal growing areas that are subject to stress conditions (Okogbenin et al., 2013).

A variety of different approaches are currently being used to minimize the negative effects of abiotic stresses on plants. Application of various stress priming agents has been presented as a potential strategy to increase biotic and abiotic stress tolerance in plants (Savvides et al., 2016). Recent studies have confirmed that the application of chemical stress priming agents represents a promising approach to manage plant stress adaptation under field conditions by activating their innate stress-adaptive mechanisms (Kinoshita and Seki, 2014).

The application of exogenous methyl jasmonate has been reported to enhance abiotic stress tolerance in soybean (Yoon et al., 2009). The application of exogenous strigolactone enhanced drought tolerance in *Arabidopsis* (Ha et al., 2014). The application of salicylic acid was shown to be beneficial for plants either in optimal or stress environments, through the regulation of various plant metabolic processes and through the modulation of the production of varied osmolytes, secondary metabolites and the plant-nutrient status, to protect plants under abiotic stress conditions (Khan et al., 2015). Chemicals such as ethanol, mandipropamid, melatonin, polyamines, and sodium nitroprusside enhanced abiotic stress tolerance in plants (Park et al., 2015; Savvides et al., 2016; Nguyen et al., 2017). The epigenetic inhibitors such as Ky-2, Ky-9, and Ky-72 increased salinity stress avoidance in *Arabidopsis* (Sako et al., 2016; Nguyen et al., 2018a,b). The *in vitro* cassava plants enhanced salinity stress tolerance by suberoylanilide hydroxamic acid (SAHA) treatment (Patanun et al., 2017). Recently, the application of acetic acid was also reported to enhance drought tolerance in a variety of plant species, including *Arabidopsis*, rice, maize, rapeseed, and wheat by activating the JA-signaling pathway (Kim et al., 2017). However, the effects of acetic acid application on increased drought tolerance in woody plants such as a tropical crop “cassava” remain elusive. In the present study, physiological- and molecular-responsive mechanisms were analyzed to elucidate the effect of acetic acid on cassava plants subjected to drought. Results of the study indicated that the acetic acid treatment increased drought avoidance in cassava. This was accomplished by the ability of acetic acid to regulate the rate of transpiration and maintain the water content in cassava leaves.

## Materials and Methods

### Cassava Cultivar and Plant Preparation

The African cassava cultivar “60444 (*Manihot esculenta* Crantz)” was obtained from the International Institute of Tropical Agriculture (IITA, Nigeria) *in vitro* cassava germplasm collection. The *in vitro* cassava plantlets were acclimated to ambient atmospheric conditions and were subsequently maintained under a greenhouse condition (50% humidity, 28°C, and 16-h supplemental lighting). Cassava plants grow well under the greenhouse condition. Stem cuttings (approximately 3 cm) were obtained from individual plants and propagated. After reaching an approximate stem length of 15 cm from the soil surface, the plants were transferred to a plastic pot (7.9 diameter × 6 height cm) filled with vermiculite. After transfer, the plants were grown under a greenhouse condition for 2 weeks and then used in the drought experiment. The treatment with 10–20 mM acetic acid solution was effective in rice, maize, wheat, rapeseed and *Arabidopsis* (Kim et al., 2017). In this study, the treatment with 10 mM acetic acid solution was performed in cassava plants because wilting leaves were observed by an application of 20, 30-, and 50-mM acetic acid solution. Also, to examine the effect of lower concentration of acetic acid on cassava plants, we also evaluated 1 mM acetic acid-treated plants with regarding to the drying test and measurement of net photosynthesis rate. The plants were watered with acetic acid or plain water (control) for 7 days, and then exposed to a drought for 14 days under a greenhouse condition to remove all water from soil pot. The phenotype of wilting leaves can be observed during 14 days of drying.

### Quantification of Leaf Wilting

Cassava plants without the application of acetic acid were placed on a rotating table, and images of the plant were taken from 360 degrees to quantify the extent of leaf wilting due to the imposed drought. Each individual cassava plant had 6–10 leaves with leaves numbered in order from the top, e.g., 1, 2,…, 10 (Supplementary Figure S1A). An image of each leaf in which the petiole was parallel to the camera was selected and analyzed. For each leaf, the midrib line was drawn between the base and the midpoint of the midrib (Supplementary Figures S1B,C). The angle made by the midrib line of the central leaflet with the vertical axis was measured using ImageJ software and used as an indicator of the level of drought or wilting (Supplementary Figures S1D,E).

### Determination of Fresh and Dry Weight, and Relative Water Content in Water- and Acetic Acid-Treated Plants

Leaves of stressed and non-stressed plants were collected separately and used to measure fresh weight and dry weight, and relative water content (RWC) using a previously described protocol (Nishiyama et al., 2011).

### Determination of Chlorophyll and Carotenoid Content

**FIGURE 1:**
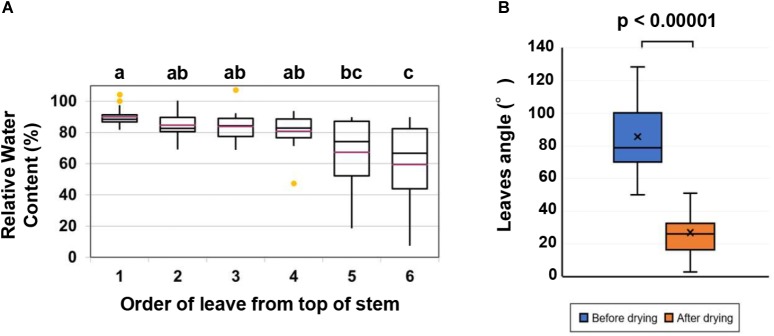
Quantification of leaf wilting and relative water content (RWC) in leaves. **(A)** RWC in the first six leaves from the top of the stem of cassava plants subjected to drought tolerance by allowing the soil to dry. Data represent the mean ± SE (*n* = 15). Different superscripted letters (a, b and c) within the column indicate statistically significant differences among the treatments determined by conducting a Scheffe multiple comparison (*P* < 0.05). **(B)** Comparison of the leaves angle before and after the drought. A paired *t*-test indicated a significant change after the drying process (*n* = 30 leaves, *p* < 0.00001).

The fifth leaf was selected for the measurement of relative water and chlorophyll contents to determine the effect of acetic acid treatment on drought. Because the RWC in cassava leaves was decreasing from the bottom to the top of stem (Figure 1A). The chlorophyll and carotenoid in approximately 0.5 g fresh weight of the fifth leaf of cassava plants were extracted by shaking (200 rpm) them overnight in the dark in 30 mL of an 80% acetone solution. Subsequently, 1.0 mL of the extracted solution was used to measure absorbance of chlorophyll at 645 nm and 663 nm for carotenoid at 470 nm in a spectrophotometer. Chlorophyll and carotenoid levels were calculated as described by Mostofa et al. (2015).

### Thermal Imaging

Thermal images were captured using an R500EX-S infrared camera equipped with a standard lens (Nippon Avionics Co., Ltd.). To measure the leaf temperature in narrow range, the camera was mounted vertically at approximately 60 cm above the leaf canopy for observations. To observe the leaf temperature in broad range, the camera adjusted with angle of approximately 50 degree was set an approximately 100 cm above leaf canopy. Thermal images were stored by interval of a minute and subsequently analyzed for temperature determination on a custom python script.

### Total RNA

For total RNA preparation, cassava plants were grown and treated with 10 mM acetic acid or water (control) for 7 days. The first to third leaves from the top of stems were collected and frozen in liquid nitrogen for total RNA purification. Frozen leaves from water- and acetic acid-treated plants were pulverized in liquid nitrogen using a Multi Beads Shocker system (Yasuikiki). Total RNA was extracted from 100 mg of leaves using the method described by Utsumi et al. (2017). Total RNA samples were subsequently purified using a Plant RNA Reagent (ThermoFisher) according to the manufacturer’s instructions, and samples of total RNA were treated with DNase I (Takara), and an RNase inhibitor, for 30 min at 37°C to eliminate genomic DNA contamination in the samples. Samples of total RNA were purified using a RNeasy Plant Mini Kit (QIAGEN) according to the manufacturer’s instructions and RNA quality was evaluated by electrophoresis using a Bioanalyzer (Agilent). After extraction, the total RNA samples were stored at -80°C until further use.

### Oligo-Microarray Analysis of Gene Expression and Statistical Analyses

Total RNA was used to evaluate gene expression using a cassava DNA oligo-microarray that included more than 30,000 probes as described by Utsumi et al. (2016). Leaves were collected from four separate water- and four 10 mM acetic acid-treated cassava plants and treated as one biological replicate. The presented gene expression data were collected from three independent biological replicates. A total of six expression microarray data sets were analyzed using GeneSpring GX (Agilent Technologies, United States). A 75-percentile normalization of the expression level was performed on all six samples. The normalized signal intensity was transformed into a log_2_ ratio for display and analysis. The normalized signal intensity of transcripts from each sample group (experiment) was used in the statistical analysis. An analysis of variance test was conducted to determine the effect of treatment (water treatment vs. 10 mM acetic acid treatment). Changes in gene expression were statistically analyzed using an unpaired *t*-test for the two treatment groups. A false discovery rate (*q*-value) was calculated by a Westfall–Young multiple testing correction based on an unpaired *t*-test. The information from the oligo-DNA microarray was deposited in the Gene Expression Omnibus (GEO) at NCBI. The accession numbers are: Platform, GPL22197; Series, GSE122140 *Manihot esculenta* microarray; Samples, GSM3455975-GSM3455980.

### Statistical Analyses

Physiological and biochemical data were analyzed using a one-way ANOVA and differences among means were analyzed by Scheffé’s method (*p* < 0.05) (StatPlus 5 pro AnalystSoft Inc., United States) or a Duncan’s multiple range test (*P* < 0.05) (using IBM SPSS software package 21.0).

### Phytohormone Measurements

Endogenous ABA, JA, and JA-Ile were extracted with 80% (v/v) acetonitrile containing 1% (v/v) acetic acid from the first to third leaves from the top of stems treated with 10 mM acetic acid or water for 7 days after freeze-drying. Hormone contents were determined using a UPLC-MS/MS system consisting of a quadrupole/time-of-fight tandem mass spectrometer (Triple TOF 5600, SCIEX, Concord, ON, Canada), and a Nexera UPLC system (Shimadzu Corp., Kyoto, Japan) by Kanno et al. (2016).

### Phylogenetic Analysis of Amino Acid Sequences

Homology between *Arabidopsis* and cassava on amino acid sequences was determined by BLASTX^1^ where the query nucleotide sequences were aligned with homologous sequences retrieved from Phytozome v12.1.6^2^. Phylogenetic trees were generated by the neighbor-joining method using CLUSTAL_W 2.0.12 software, and branch significance was analyzed by bootstrap with 1,000 replicates. Phylogenic trees were visualized using GENTYX-Tree 2.2.5 (Genetyx) software.

## Results

### Relationship Between Angle of Leaf Wilting and Water Loss in Leaves

As the water from the soil in the potted cassava plants decreased, cassava leaves wilted and dropped from the stem. The RWC of leaves gradually declined from the top leaf downward after drying (Figure 1A). While the majority of the leaves face upward, however, the angle between the midrib and the vertical axis did not correctly indicate the leaflet angle in leaves which faced the front of the camera. Therefore, images in which the width of the leaflet exceeded 10% of the length of the midrib were excluded from the analyzed dataset. For example, the width of the leaflet in Supplementary Figure S1D is 5.2% of the midrib length, but 19.9% in Supplementary Figure S1E. In this case, the latter leaf was not used in the analyzed dataset. The uppermost leaves were too small to measure the angle accurately, and the lower leaves which wilted due to the drought often rolled up and the angle was also unable to be measured. Thus, only leaves 2 through 5 were used in the analysis (Supplementary Figure S1A). The angles of 30 leaves before and after imposed drought were analyzed. The angle between the midrib and the vertical axis decreased significantly in response to the drought. The average degree angle changed from 85.6 to 26.9 (Figure 1B).

### Effect of the Acetic Acid Treatment on Leaf Wilting and Leaf Water Status

**FIGURE 2:**
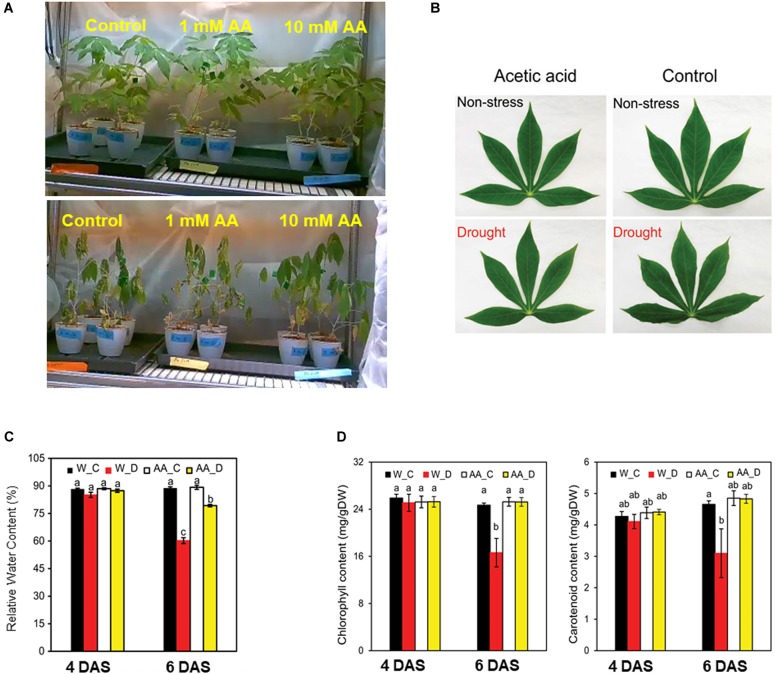
**(A)** Representative cassava plants that were either treated with 1- or 10-mM acetic acid or water-treated (control) at 9 days after the initiation of soil drying (bottom) and prior to drying (upper). **(B)** Representative leaf from 10 mM acetic acid-treated and water-treated (control) cassava plants at 4 and 6 days after the commencement of soil drying. **(C)** Relative water content (RWC) of the fifth leaf of cassava plants at 4 and 6 days after the commencement of the soil drying treatment. Data represent the mean ± SE (*n* = 4). **(D)** Chlorophyll and carotenoid levels of the fifth leaf of cassava plants at 4 and 6 days after the commencement of the soil drying treatment. Data represent the mean ± SE (*n* = 4). Different superscripted letters (a, b, and c) within a column in **(C,D)** indicate statistically significant differences among the treatments as determined by a Duncan’s multiple range test (*P* < 0.05).

To determine the effect of acetic acid treatment on drought in cassava, the fifth leaf was selected for the measurement of relative water and chlorophyll contents because the RWC in cassava leaves decreased from the bottom to the top of the stem (Figure 1A). Results indicated that the acetic acid-treated plants exhibited a less wilted leaf phenotype in response to the drought, relative to the water-treated, control plants (Figure 2A). To further investigate the response of acetic acid-treated cassava plants to drought, RWC was measured. As illustrated in Figure 2B,C, RWC was unchanged under non-stress conditions in the acetic acid-treated group relative to the water control group. In response to the drought imposed by the drying soil treatment, however, the RWC of leaves in the acetic acid-treated cassava plants was significantly higher than in the water-treated plants at 6 days, but not 4 days, after the onset of the drought (Figure 2C). These results suggest that the enhancement of drought avoidance induced by the acetic acid treatment is associated with maintaining the water status in plants.

### Decreased Degradation of Chlorophyll and Carotenoid in Acetic Acid-Treated Plants Subjected to Drought

The imposed drought resulted in a considerable decline in the level of total chlorophyll (Chl) and carotenoids. Total Chl and carotenoid levels, however, were higher in acetic acid-treated plants than in water-treated plants subjected to drought (Figure 2D). These results suggest that the negative effect of drought on photosynthetic pigments and carotenoids were substantially minimized by the acetic acid treatment. The level of total Chl and carotenoids was unchanged under non-stressed conditions and after 4 days of drought. After 6 days of drought, however, total Chl and carotenoid levels decreased in the water-treated control plants but not in the acetic acid-treated plants (Figure 2D). These results are consistent with the RWC and leaf wilting phenotype (Figure 2B,C) data obtained in response to the drying soil treatment.

### The Acetic Acid Treatment Increases Leaf Temperature and Decreases Net Photosynthesis and Stomatal Conductance

**FIGURE 3:**
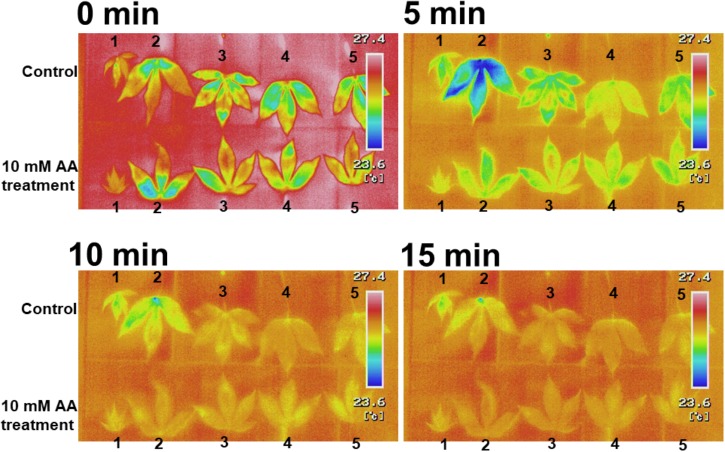
Thermal images of detached leaves from cassava plants treated with 10 mM acetic acid or water (control). Cassava plants were treated with 10 mM acetic acid or water for 7 days. The first six leaves from the top stem were removed and the leaf temperature was measured at 0, 5, 10, and 15 min after leaf removal.

**FIGURE 4:**
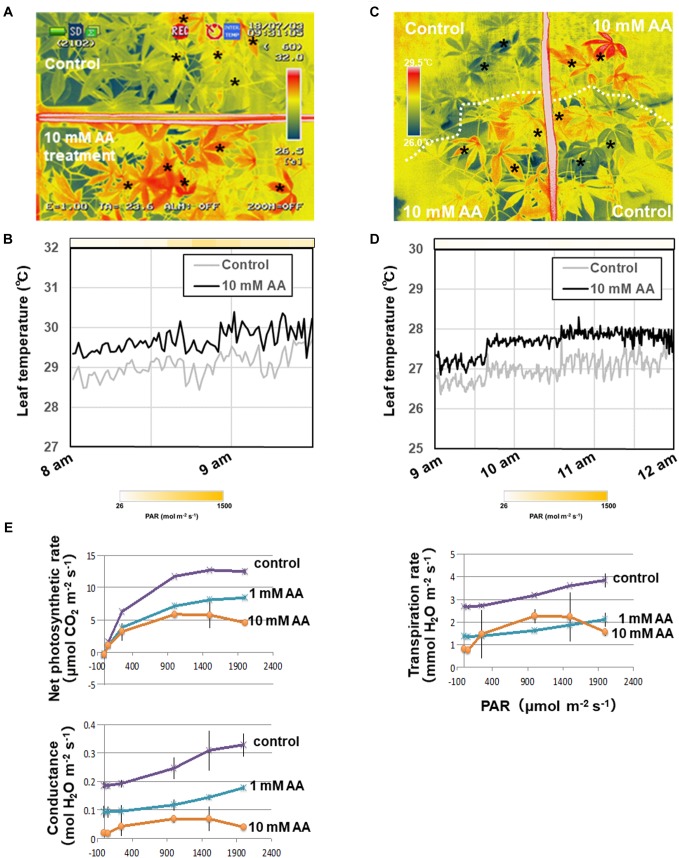
Thermal images of whole plants and the net photosynthetic rate of cassava plants treated with either 10 mM acetic acid or water (control). **(A)** Thermal images of cassava plants treated with either 10 mM acetic acid or water (control) after 3 days under sunlight. **(B)** Leaf temperature differences in cassava plants treated with either 10 mM acetic acid or water (control) and placed in the sunlight. The mean leaf temperature was calculated based on temperature of five places on each leaf indicated by asterisks in **(A)**. The color gradation on the bar indicates the photosynthetically active radiation (PAR). **(C)** Thermal images of cassava plants treated with either 10 mM acetic acid or water (control) after 5 days under the cloudy condition. **(D)** Differences in leaf temperature of cassava plants treated with either 10 mM acetic acid or water (control) and placed under the cloudy condition. The mean leaf temperature was calculated based on the temperature of five places on each leaf indicated by asterisks in **(C)**. The color gradation on the bar indicates the PAR. **(E)** Net photosynthetic rate, stomatal conductance and transpiration rate of first to third leaves from the top of the stem of cassava plants treated with 10 mM acetic acid or water (control) at 7 days after the commencement of the drought. Mean ± SD of nine leaves from three plants.

The acetic acid treatment delayed leaf wilting in cassava plants subjected to drought. To determine the physiological effect of the acetic acid treatment on leaves, leaf temperature and gas exchange in acetic acid-treated and non-treated plants was measured. Leaf temperature in detached acetic acid-treated leaves rapidly increased in comparison to the temperature in detached water-treated leaves (Figure 3). Leaf temperatures within intact, acetic acid-treated plants were measured under sunny (Figure 4A,B) or cloudy conditions (Figure 4C,D). The acetic acid-treated plants exhibited a higher leaf temperature than leaves in water-treated plants (Figure 4A,C). Leaf temperatures in acetic acid-treated plants under sunny conditions were also continuously higher than in leaves of water-treated plants (Figure 4B,D). Leaf temperature under the high photosynthetically active radiation (PAR) of a sunny day increased more significantly than under the low PAR of a cloudy day (Figure 4B,D). The net photosynthesis rate, stomatal conductance and transpiration rate in acetic acid-treated plants decreased considerably after 7 days of the acetic acid treatment in comparison to control (Figure 4E). We also examined the net photosynthetic rate, conductance and transpiration rate with 1 mM or 10 mM acetic acid treated-plants. The net photosynthetic rate, conductance and transpiration rate in 10 mM acetic acid-treated plants were decreased to 40%, 15%, and 38%, respectively, in comparison to that in control under 2,000 μmol mol^-2^ s^-1^. In 1 mM acetic acid treated-plants, the values were kept at 64%, 58%, and 50%, respectively (Figure 4E). These results suggest that acetic acid might induce stomatal closure and the degree of physiological effect to acetic acid might depend on the concentration of acetic acid used.

### Increase of Abscisic Acid (ABA) Level and Expression of ABA Signaling Pathway Genes in Leaves of Acetic Acid-Treated Cassava Plants at 7 Days After the Imposition of a Drought

**FIGURE 5:**
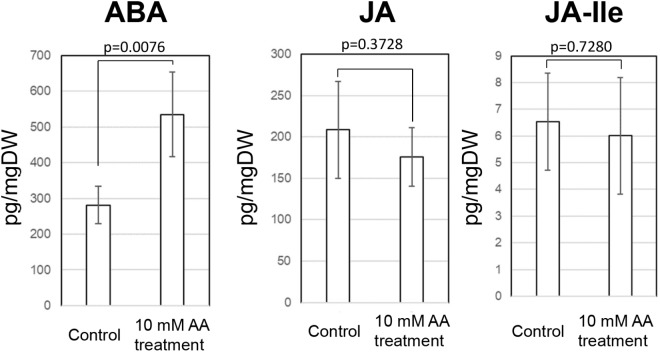
ABA, JA, and JA-Ile contents in the cassava leaves at 7 days after external application of acetic acid and water (control). A *p*-value on figure was measured by paired *t*-test.

**FIGURE 6:**
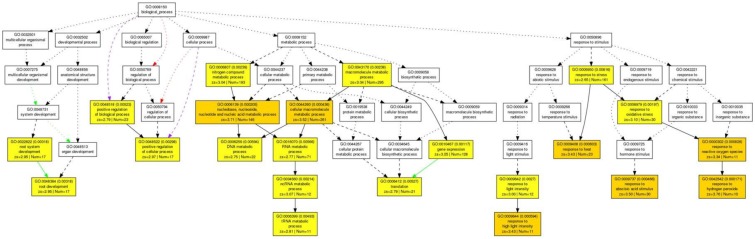
Parametric analysis of gene set enrichment (PAGE) in the biological process of 1,945 genes in cassava plants treated with 10 mM acetic acid in comparison to cassava plants treated with water treatment as determined with agriGO software. The genes that were annotated based on homology with *Arabidopsis* were selected by Westfall-Young multiple testing correction (FDR < 0.0001). The first pair of numerals represents the number of genes in the input list associated with the GO term and the number of genes in the input list. The second pair of numerals represents the number of genes associated with the GO term in the *Arabidopsis* database (TAIR10), the total number of *Arabidopsis* genes with GO annotations in TAIR10 and the *p*-value in parenthesis. Box colors in PAGE indicate levels of statistical significance: yellow < 0.01; orange < 0.001. White boxes are shown as non-significant terms.

Acetic acid-treatment delayed the decrease in RWC in leaves of cassava plants exposed to a drought. In *Arabidopsis*, research on the effect of acetic acid has suggested that acetic acid operates through jasmonate (JA) signaling to respond to drought and maintains the RWC in plants. Therefore, to investigate the effect of the acetic acid, the measurement of ABA, JA and jasmonate-Ile (JA-Ile) and transcriptome analysis of acetic acid-treated- and control leaves were conducted. The ABA content from 10 mM acetic acid-treated leaves was significantly increased 1.9 folds in comparison to control one but JA and JA-Ile contents were not changed (Figure 5). In the transcriptome analysis, results indicated that there was an upregulation of genes involved in the ABA signaling pathway and heat shock proteins (HSPs) in the acetic acid-treated leaves relative to control leaves of cassava plants (Table 1). The microarray analysis identified 2,399 genes that showed significant differences (FDR < 0.0001) in expression as determined with Westfall–Young multiple testing correction based on an unpaired *t*-test. Among the differentially expressed genes, 227 and 330 genes were twofold up- or down-regulated, respectively, by the acetic acid treatment. Functional category classification using the gene ontology (GO) of the *Arabidopsis* Information Resource (TAIR10) for Biological Process by agriGO was performed on the 1,945 of the differentially expressed cassava genes that could be annotated based on the genome from *Arabidopsis thaliana*. These genes were classified into the GO terms “response to abscisic acid stimulus,” “response to hydrogen peroxide,” “response to heat,” “response to high light intensity,” “tRNA metabolic process,” and “positive regulation of cellular process” for biological process (Figure 6). Here, we focused on “response to abscisic acid stimulus,” “response to hydrogen peroxide,” “response to heat,” “response to high light intensity” due to show the increase of ABA level in acetic acid-treated leaves. The stress-upregulated cassava genes classified in these GO terms include the homolog of a key gene involved in response to stress and ABA; *ABA INSENSITIVE 2, ABI2*(AT5G57050.1), which encodes a member of a *protein phosphatase 2C* (*PP2C*) (Table 1) that plays a role in ABA signal transduction as negative regulators (Leung et al., 1997); several *HSPs*, such as *HSP21* (AT4G27670*.1*), *HSP70* (AT3G12580.1), *HSP18.2* (AT5G59720.1), and *HSP20* (AT1G54050.1 and AT2G29500.1), which play important roles in cells by preventing aggregation of destabilized proteins exposed to high heat and light intensity (Basha et al., 2012); *OPEN STOMATA 1, OST1* (AT4G33950.2), which encodes the ABA-activated protein kinase, a member of *SNF1-related protein kinases* (*SnRK2*) that are involved in stomatal closure through ABA induction (Umezawa et al., 2010) (Supplementary Figure S2A); *ABI five binding protein 2* (*AFP2*) (AT1G13740.1), whose members interact with the transcription factor, *ABA-Insensitive5* (*ABI5*) which is mediated in ABA response (Lynch et al., 2017); and two different kinds of *PP2C* genes (AT5G51760.1 and AT5G59220.1) that may play a role in ABA signal transduction along with ABI2.

**Table 1:** Acetic acid-responsive genes in cassava.

Encoded proteins/other features ^a^	Log_2_ ratio^b^	AGI code ^c^	*E*-value	Cassava gene model ^d^
**Genes that respond to high light intensity (GO:0009644)**				
Heat shock protein 21, HSP21	2.5	AT4G27670.1	4.2E-45	Manes.01G042200
Ethylene-dependent gravitropism-deficient and yellow-green-like 3	2.0	AT1G17870.1	1.4E-45	Manes.10G087000
ABA INSENSITIVE 2, ABI2	1.9	AT5G57050.1	3E-11	Manes.06G158800
Heat shock protein 70 (Hsp 70) family protein	1.9	AT3G12580.1	0	Manes.11G067500
Heat shock protein 18.2	1.2	AT5G59720.1	3E-19	Manes.04G138400
Stress-inducible protein, HOP3	0.8	AT4G12400.2	0	Manes.02G035100
HSP20-like chaperones superfamily protein	0.7	AT1G54050.1	1.4E-45	
HSP20-like chaperones superfamily protein	0.6	AT2G29500.1	0	Manes.02G124600
Fatty acid desaturase 5	-0.4	AT3G15850.1	2E-38	Manes.04G112800
Proton gradient regulation 5	-0.7	AT2G05620.1	7E-43	Manes.03G160100
**Genes that respond to heat (GO:0009408)**				
Heat shock protein 21, HSP21	2.5	AT4G27670.1	4.2E-45	Manes.01G042200
Ethylene-dependent gravitropism-deficient and yellow-green-like 3	2.0	AT1G17870.1	1.4E-45	Manes.10G087000
ABA INSENSITIVE 2, ABI2	1.9	AT5G57050.1	3E-11	Manes.06G158800
Heat shock protein 70 (Hsp 70) family protein	1.9	AT3G12580.1	0	Manes.11G067500
Multiprotein bridging factor 1C, MBF1C	1.2	AT3G24500.1	0	Manes.03G019200
Heat shock protein 18.2	1.2	AT5G59720.1	3E-19	Manes.04G138400
5′–3′ exonuclease family protein	0.9	AT3G28030.1	0.0000006	Manes.15G181100
Ubiquitin-conjugating enzyme 5	0.9	AT5G41340.1	0	Manes.02G021200
Stress-inducible protein, HOP3	0.8	AT4G12400.2	0	Manes.02G035100
HSP20-like chaperones superfamily protein	0.7	AT1G54050.1	1.4E-45	
Cytochrome P450, family 71	0.7	AT1G13080.1	3E-38	Manes.14G059200
Regulatory protein (NPR1)///unknown	0.6	AT1G64280.1	2E-15	Manes.16G100600
HSP20-like chaperones superfamily protein	0.6	AT2G29500.1	0	Manes.02G124600
Heat shock protein 90.1	0.6	AT5G52640.1	0	Manes.06G152800
Aldehyde dehydrogenase 5F1	0.6	AT1G79440.1	0	
Molecular chaperone Hsp40/DnaJ family protein	0.5	AT1G80030.3	0	Manes.11G058000
Serine/threonine protein kinase 2	-0.4	AT3G08720.2	0	
DNAJ heat shock N-terminal domain-containing protein	-0.4	AT3G08970.1	0	Manes.08G094300
DNAJ heat shock family protein	-0.5	AT2G22360.1	1E-29	Manes.14G168300
Chitinase family protein	-0.6	AT1G05850.1	2E-14	Manes.05G195100
Heat shock cognate protein 70-1	-0.7	AT5G02500.2	0	Manes.10G031400
Heat shock protein 17.4	-0.7	AT3G46230.1	0	Manes.10G020000
**Genes that respond to abscisic acid stimulus (GO:0009737)**				
TSPO (outer membrane tryptophan-rich sensory protein)-related	3.3	AT2G47770.1	0	Manes.05G063900
ABA INSENSITIVE 2, ABI2	1.9	AT5G57050.1	3E-11	Manes.06G158800
Drought-induced 21	1.6	AT4G15910.1	3E-14	
Homeobox 12	1.5	AT3G61890.1	0	
Protein phosphatase 2C family protein, AHG1	1.3	AT5G51760.1	5E-25	
Enolase	1.3	AT2G36530.1	6E-41	Manes.14G033200
Fructose-bisphosphate aldolase 1///fructose-bisphosphate aldolase 2	1.2	AT4G38970.1	7E-37	
Cold regulated 413 plasma membrane 1	1.2	AT2G15970.1	7E-20	
Protein phosphatase 2C family protein	1.0	AT5G59220.1	1E-12	Manes.02G128000
Catalase 1	1.0	AT1G20630.1	1E-41	
Pentatricopeptide (PPR) domain protein 40	0.9	AT3G16890.1	0	Manes.03G202100
Myb domain protein 60	0.9	AT1G08810.1	2E-10	
OPEN STOMATA 1, OST1	0.9	AT4G33950.2	7E-19	Manes.15G143700
ABI five binding protein 2, AFP2	0.7	AT1G13740.1	0.0000005	Manes.01G027200
Indole-3-butyric acid response 5	0.7	AT2G04550.3	6E-15	Manes.13G064900
G-box binding factor 3	0.6	AT2G46270.1	4E-37	
Multiprotein bridging factor 1C, MBF1C	0.5	AT3G24500.1	5E-18	Manes.03G019200
CBL-interacting protein kinase 15	0.5	AT5G01810.3	9E-30	
Prenyltransferase family protein	0.4	AT5G40280.1	0.0000002	
Abscisic acid responsive elements-binding factor 2	0.4	AT1G45249.1	5E-44	Manes.05G174500
Phosphatidic acid phosphatase (PAP2) family protein	-0.4	AT3G58490.2	1E-23	Manes.08G040500
RING-H2 finger A2A	-0.4	AT1G15100.1	1E-41	Manes.06G063700
RAC-like 10	-0.5	AT3G48040.1	0	
Homeodomain-like superfamily protein	-0.5	AT1G01060.5	2E-29	
Homeodomain-like superfamily protein	-0.6	AT3G09600.1	3E-19	
Unknown protein	-0.6	AT5G25610.1	0	Manes.03G126100
HVA22 homolog E	-0.6	AT5G50720.1	5E-44	Manes.14G049000
MIRO-related GTP-ase 2	-1.0	AT3G63150.1	0	
RGA-like 2	-1.2	AT3G03450.1	0	Manes.05G004100
**Genes that respond to hydrogen peroxide (GO:0042542)**				
Heat shock protein 21	2.5	AT4G27670.1	4.20E-45	Manes.01G042200
Heat shock protein 70 (Hsp 70)	1.9	AT3G12580.1	0	Manes.11G067500
Purple acid phosphatase 17	1.5	AT3G17790.1	6.00E-33	Manes.14G173500
Ethylene-dependent gravitropism-deficient and yellow-green-like 3	1.4	AT1G17870.1	0	Manes.10G087000
Heat shock protein 18.2	1.2	AT5G59720.1	3.00E-19	Manes.04G138400
Catalase 1	1.0	AT1G20630.1	1.00E-41	
HSP20-like chaperones superfamily protein	0.7	AT1G54050.1	1.40E-45	
Catalase 2	0.6	AT4G35090.1	0	
HSP20-like chaperones superfamily protein	0.6	AT2G29500.1	0	Manes.02G124600


*^*a*^Encoded proteins/other features indicate the putative functions of the gene products that are expected from sequence similarity. The information for the NCBI protein reference sequence with the highest sequence similarity with the probes is shown. ^*b*^Log_*2*_ ratio = log_*2*_ (10 mM acetic acid/control). ^*c*^The information for AGI locus ID with the highest sequence similarity with the probe is shown. ^*d*^The information for cassava gene code with the highest sequence similarity with the probe is shown.*

Although several genes related to the ABA signaling pathway were identified, the expression of key genes in the ABA biosynthetic pathway, such as the *9-cis-epoxycarotenoid dioxygenases* (*NCEDs*), were not dramatically changed in response to the acetic acid (Supplementary Figure S2B and Supplementary Table S1) (Iuchi et al., 2001). Upregulation of JA biosynthetic genes, such as *allene oxide cyclases* (*AOCs*), and signaling pathway genes, such as *COI1*, *JAZ*, and *MYC* families, was also not observed in cassava plants treated with acetic acid (Supplementary Table S2). In the current study of cassava, the expression of genes involved in the ABA signaling pathway were upregulated by the acetic acid treatment and sunlight, suggesting that activation of the ABA signaling pathway may play a role in acetic acid-mediated drought avoidance in cassava plants through the increased ABA level.

## Discussion

Results of the present study indicate that an application of acetic acid enhances drought avoidance in cassava. Analyses of microarray experiments revealed that acetic acid upregulates the expression of ABA signaling pathway-related genes, such as *ABA-insensitive 2* (*ABI2;* Finkelstein and Somerville, 1990) and *OST1* (Mustilli et al., 2002) homologs, and stress response and tolerance-related genes, such as HSPs (Figure 5 and Table 1). Acetic acid treatment increased leaf temperature and accumulation of ABA and decreased stomatal conductance and transpiration rates; suggesting that the acetic acid treatment retained the RWC in cassava plants subjected to drought through the ABA-mediated control of stomatal aperture.

Previous studies have reported that treatment of plants with acetic acid enhances drought tolerance in *Arabidopsis*, rice, maize, wheat, and rapeseed and that activation of JA signaling is a key event in the enhancement of drought tolerance in *Arabidopsis* (Kim et al., 2017). Activation of ABA biosynthesis and the ABA signaling pathway was not observed in *Arabidopsis* in response to an acetic acid treatment (Kim et al., 2017); although the expression of ABA signaling-related genes, such as *ABI2*, *OST1*, *ABA-hypersensitive germination 1* (*AHG1*; Nishimura et al., 2007), *ABA-insensitive five-binding protein 2* (*AFP2*; Lynch et al., 2017) were upregulated in cassava in response to an acetic acid treatment (Table 1). In the present study, increased expression of JA biosynthesis and signaling pathway genes was not observed in response to the acetic acid treatment, although the expression of *JA biosynthetic enzyme AOC3* was upregulated and the level of JA increased transiently in response to an acetic acid treatment in *Arabidopsis* (Kim et al., 2017). Differences in the molecular response of cassava vs. *Arabidopsis* may be due to the following reasons: (1) innate differences in the response of woody plants, such as cassava, and herbaceous plants, such as *Arabidopsis*, to the exogenous application of acetic acid; (2) use of different experimental conditions in the studies conducted in *Arabidopsis* vs. cassava. For example, high light (in the range of 100–1,300 μmol m^-2^s^-1^) vs. low (∼100 μmol m^-2^ s^-1^) light intensity was used in studies on cassava vs. *Arabidopsis*, respectively (Kim et al., 2017; Rasheed et al., 2018).

The application of acetic acid increased the expression of various stress response and stress tolerance-related genes, such as heat shock family proteins, *HSP20* (Muthusamy et al., 2017), *HSP21* (Zhong et al., 2013), *HSP70* (Tang et al., 2016; Leng et al., 2017) and *HSP90* (Krishna and Gloor, 2001), ER stress-related gene, *HOP3* (At4g12400; Fernández-Bautista et al., 2017a,b), *catalase 1* and *catalase 2* (Zou et al., 2015), *multiprotein-bridging factor 1c* gene (*MBF1c*; Alavilli et al., 2017), and an outer membrane tryptophan-rich sensory protein (TSPO) (Guillaumot et al., 2009). HSP20 and HSP70 family members function as molecular chaperones and help plants adapt to environmental stress (Tang et al., 2016; Leng et al., 2017; Muthusamy et al., 2017). HSP21 is involved in the protection against oxidative stress (Zhong et al., 2013). HOP3, a member of the HOPs [heat shock protein 70 (HSP70) and heat shock protein 90 (HSP90) organizing proteins] family in *Arabidopsis* plays an essential role in the response of the endoplasmic reticulum (ER) in plants to abiotic stress (Fernández-Bautista et al., 2017a). HOP3 has been suggested, through its role in the alleviation of ER stress, to play an important function in plant response to various stresses (Fernández-Bautista et al., 2017a). It is plausible to suggest that acetic acid treatment enhances drought avoidance in cassava through the regulation of the ER stress response. Overexpression of *P. alpinum multiprotein-bridging factor 1c* gene (*PaMBF1c*) enhanced salt tolerance in *Arabidopsis* (Alavilli et al., 2017). Catalase plays an important role in ABA- and H_2_O_2_-mediated signal transduction and in maintaining H_2_O_2_ homeostasis in response to drought (Zou et al., 2015). *TSPO* expression is upregulated by drought in both shoots and roots, and the *TSPO* promoter has been shown to be useful for the genetic engineering of drought tolerant plants to drought (Rasheed et al., 2018).

## Conclusion

Our study demonstrates that the external application of acetic acid under high PAR leads to stomatal closure which is induced by the activation of the ABA-dependent stress response pathway in cassava, by increasing the ABA level. Our study also indicates the possibility of avoiding drought without the inhibition of plant growth, through the external application of acetic acid with a lower concentration. Although the detailed mechanisms underlying acetic acid-mediated enhancement of drought avoidance in cassava remain to be elucidated, the findings of the current study suggest that treatment of plants with simple, easily available and low cost compounds, such as acetic acid, may have beneficial effects on the growth of plants subjected to drought.

## Author Contributions

YU and MoS conceptualized the study. YU, CU, MT, CH, and ST measured the physiological parameters. TM and SM measured the leaf angle. YK and MiS performed the phytohormone measurements. YU, MT, and AM performed the microarray analysis. CU, YO, and EM performed the propagation of plants. YU, CH, and MoS wrote the original draft.

## Conflict of Interest Statement

The authors declare that the research was conducted in the absence of any commercial or financial relationships that could be construed as a potential conflict of interest.

## Footnotes

1 http://blast.st-va.ncbi.nlm.nih.gov/Blast.cgi

2 https://phytozome.jgi.doe.gov/pz/portal.html

## Supplementary Material

Figure S1 Leaf wilting phenotype of representative water-treated (control) cassava plant after being subjected to a drought **(A)**, representative cassava plant not subjected to a drought (soil drying) **(B,D,E)**, representative cassava plant subjected to a drought stress **(C)**. Images in which the width of the leaflet (blue line) exceeded 10% of the midrib length (red line) were excluded from the analyzed dataset.

Figure S2 Phylogenetic tree of SnRK2 and NCED amino acid sequences from cassava (*Manihot esculenta*, Me) and *Arabidopsis* (*Arabidopsis thaliana*, At). **(A)** SnRK2 and **(B)** NCED amino acid sequences. The distance of the branches denotes the bootstrap majority consensus values on 1,000 replicates.

Table S1 Expression of *NCED* genes that play a key role in ABA biosynthesis.

Table S2 Expression data of JA signaling pathway genes, such as *COI1*, *MYCs*, *JAZs*, and JA biosynthesis pathway genes, such as *AOCs.*

## References

[B1] Aina O. O. Dixon A. G. O. Akinrinde E. A. (2007). Effect of soil moisture stress on growth and yield of cassava in Nigeria. *Pak. J. Biol. Sci.* 10 3085–3090. 10.3923/pjbs.2007.3085.3090 19090103

[B2] Alavilli H. Lee H. Park M. Lee B. H. (2017). Antarctic moss multiprotein bridging factor 1c overexpression in *Arabidopsis* resulted in enhanced tolerance to salt stress. *Front. Plant Sci.* 8:1206. 10.3389/fpls.2017.01206 28744295 PMC5504242

[B3] Basha E. O’neill H. Vierling E. (2012). Small heat shock proteins and alpha-crystallins: dynamic proteins with flexible functions. *Trends Biochem. Sci.* 37 106–117. 10.1016/j.tibs.2011.11.005 22177323 PMC3460807

[B4] Chemonges M. Balyejusa E. K. Bisikwa J. Osiru D. S. O. (2013). Phenotypic and physiological traits associated with drought tolerant cassava cultivars in Uganda. *Afr. Crop Sci. Conf. Proceed.* 11 463–469. 10.1093/aobpla/plt007 23519782 PMC3604649

[B5] De Oliveira E. J. Morgante C. V. De Tarso Aidar S. De Melo Chaves A. R. Antonio R. P. Cruz J. L. (2017). Evaluation of cassava germplasm for drought tolerance under field conditions. *Euphytica* 213:188 10.1007/s10681-017-1972-7

[B6] El-Sharkawy M. A. (2007). Physiological characteristics of cassava tolerance to prolonged drought in the tropics: implications for breeding cultivars adapted to seasonally dry and semiarid environments. *Braz. J. Plant Physiol.* 19 257–286. 10.1590/s1677-04202007000400003

[B7] Fahad S. Bajwa A. A. Nazir U. Anjum S. A. Farooq A. Zohaib A. (2017). Crop production under drought and heat stress: plant responses and management options. *Front. Plant Sci.* 8:1147. 10.3389/fpls.2017.01147 28706531 PMC5489704

[B8] Fernández-Bautista N. Fernandez-Calvino L. Munoz A. Castellano M. M. (2017a). HOP3 a new regulator of the ER stress response in *Arabidopsis* with possible implications in plant development and response to biotic and abiotic stresses. *Plant Signal. Behav.* 12:e1317421. 10.1080/15592324.2017.1317421 28426278 PMC5501236

[B9] Fernández-Bautista N. Fernandez-Calvino L. Munoz A. Castellano M. M. (2017b). HOP3, a member of the HOP family in *Arabidopsis*, interacts with BiP and plays a major role in the ER stress response. *Plant Cell Environ.* 40 1341–1355. 10.1111/pce.12927 28155228

[B10] Finkelstein R. R. Somerville C. R. (1990). Three classes of abscisic acid (ABA)-insensitive mutations of *Arabidopsis* define genes that control overlapping subsets of ABA responses. *Plant Physiol.* 94 1172–1179. 10.1104/pp.94.3.1172 16667813 PMC1077358

[B11] Fu L. Ding Z. Han B. Hu W. Li Y. Zhang J. (2016). Physiological investigation and transcriptome analysis of polyethylene glycol (PEG)-induced dehydration stress in cassava. *Int. J. Mol. Sci.* 17:283. 10.3390/ijms17030283 26927071 PMC4813147

[B12] Guillaumot D. Guillon S. Deplanque T. Vanhee C. Gumy C. Masquelier D. (2009). The *Arabidopsis* TSPO-related protein is a stress and abscisic acid-regulated, endoplasmic reticulum-Golgi-localized membrane protein. *Plant J.* 60 242–256. 10.1111/j.1365-313X.2009.03950.x 19548979

[B13] Ha C. V. Leyva-Gonzalez M. A. Osakabe Y. Tran U. T. Nishiyama R. Watanabe Y. (2014). Positive regulatory role of strigolactone in plant responses to drought and salt stress. *Proc. Natl. Acad. Sci. U.S.A.* 111 851–856. 10.1073/pnas.1322135111 24379380 PMC3896162

[B14] Iuchi S. Kobayashi M. Taji T. Naramoto M. Seki M. Kato T. (2001). Regulation of drought tolerance by gene manipulation of 9-cis-epoxycarotenoid dioxygenase, a key enzyme in abscisic acid biosynthesis in *Arabidopsis*. *Plant J.* 27 325–333. 10.1046/j.1365-313x.2001.01096.x 11532178

[B15] Jolayemi O. L. Opabode J. T. (2018). Responses of cassava (Manihot esculenta Crantz) varieties to in vitro mannitol-induced drought stress. *J. Crop Improv.* 32 566–578. 10.1080/15427528.2018.1471431

[B16] Kamanga R. M. Mbega E. Ndakidemi P. (2018). Drought tolerance mechanisms in plants: physiological responses associated with water deficit stress in Solanum lycopersicum. *Adv. Crop Sci. Technol.* 6:3 10.4172/2329-8863.1000362

[B17] Kanno Y. Oikawa T. Chiba Y. Ishimaru Y. Shimizu T. Sano N. (2016). AtSWEET13 and AtSWEET14 regulate gibberellin-mediated physiological processes. *Nat. Commun.* 7:13245. 10.1038/ncomms13245 27782132 PMC5095183

[B18] Khan M. I. Fatma M. Per T. S. Anjum N. A. Khan N. A. (2015). Salicylic acid-induced abiotic stress tolerance and underlying mechanisms in plants. *Front. Plant Sci.* 6:462. 10.3389/fpls.2015.00462 26175738 PMC4485163

[B19] Kim J. M. To T. K. Matsui A. Tanoi K. Kobayashi N. I. Matsuda F. (2017). Acetate-mediated novel survival strategy against drought in plants. *Nat. Plants* 3:17097. 10.1038/nplants.2017.97 28650429

[B20] Kinoshita T. Seki M. (2014). Epigenetic memory for stress response and adaptation in plants. *Plant Cell Physiol.* 55 1859–1863. 10.1093/pcp/pcu125 25298421

[B21] Krishna P. Gloor G. (2001). Hsp90 family of proteins in *Arabidopsis thaliana*. *Cell Stress Chaperones* 6 238–246. 11599565 10.1379/1466-1268(2001)006<0238:thfopi>2.0.co;2 PMC434405

[B22] Leng L. Liang Q. Jiang J. Zhang C. Hao Y. Wang X. (2017). A subclass of HSP70s regulate development and abiotic stress responses in *Arabidopsis thaliana*. *J. Plant Res.* 130 349–363. 10.1007/s10265-016-0900-6 28004282

[B23] Leung J. Merlot S. Giraudat J. (1997). The *Arabidopsis* ABSCISIC ACID-INSENSITIVE2 (ABI2) and ABI1 genes encode homologous protein phosphatases 2C involved in abscisic acid signal transduction. *Plant Cell* 9 759–771. 10.1105/tpc.9.5.759 9165752 PMC156954

[B24] Lynch T. J. Erickson B. J. Miller D. R. Finkelstein R. R. (2017). ABI5-binding proteins (AFPs) alter transcription of ABA-induced genes via a variety of interactions with chromatin modifiers. *Plant Mol. Biol.* 93 403–418. 10.1007/s11103-016-0569-1 27942958

[B25] Mostofa M. G. Rahman A. Ansary M. M. Watanabe A. Fujita M. Tran L. S. (2015). Hydrogen sulfide modulates cadmium-induced physiological and biochemical responses to alleviate cadmium toxicity in rice. *Sci. Rep.* 5:4078. 10.1038/srep14078 26361343 PMC4566128

[B26] Mustilli A. C. Merlot S. Vavasseur A. Fenzi F. Giraudat J. (2002). *Arabidopsis* OST1 protein kinase mediates the regulation of stomatal aperture by abscisic acid and acts upstream of reactive oxygen species production. *Plant Cell* 14 3089–3099. 10.1105/tpc.007906 12468729 PMC151204

[B27] Muthusamy S. K. Dalal M. Chinnusamy V. Bansal K. C. (2017). Genome-wide identification and analysis of biotic and abiotic stress regulation of small heat shock protein (HSP20) family genes in bread wheat. *J. Plant Physiol.* 211 100–113. 10.1016/j.jplph.2017.01.004 28178571

[B28] Nguyen H.-C. Lin K.-H. Ho S.-L. Chiang C.-M. Yang C.-M. (2018a). Enhancing the abiotic stress tolerance of plants: from chemical treatment to biotechnological approaches. *Physiol. Plant* 164 452–466. 10.1111/ppl.12812 30054915

[B29] Nguyen H. M. Sako K. Matsui A. Ueda M. Tanaka M. Ito A. (2018b). Transcriptomic analysis of *Arabidopsis thaliana* plants treated with the Ky-9 and Ky-72 histone deacetylase inhibitors. *Plant Signal. Behav.* 13:e1448333. 10.1080/15592324.2018.1448333 29517946 PMC5927655

[B30] Nguyen H. M. Sako K. Matsui A. Suzuki Y. Mostofa M. G. Ha C. V. (2017). Ethanol enhances high-salinity stress tolerance by detoxifying reactive oxygen species in *Arabidopsis thaliana* and rice. *Front. Plant Sci.* 8:1001. 10.3389/fpls.2017.01001 28717360 PMC5494288

[B31] Nishimura N. Yoshida T. Kitahata N. Asami T. Shinozaki K. Hirayama T. (2007). ABA-hypersensitive germination1 encodes a protein phosphatase 2C, an essential component of abscisic acid signaling in *Arabidopsis* seed. *Plant J.* 50 935–949. 10.1111/j.1365-313X.2007.03107.x 17461784

[B32] Nishiyama R. Watanabe Y. Fujita Y. Le D. T. Kojima M. Werner T. (2011). Analysis of cytokinin mutants and regulation of cytokinin metabolic genes reveals important regulatory roles of cytokinins in drought, salt and abscisic acid responses, and abscisic acid biosynthesis. *Plant Cell* 23 2169–2183. 10.1105/tpc.111.087395 21719693 PMC3160038

[B33] Okogbenin E. Setter T. L. Ferguson M. Mutegi R. Ceballos H. Olasanmi B. (2013). Phenotypic approaches to drought in cassava: review. *Front. Physiol.* 4:93. 10.3389/fphys.2013.00093 23717282 PMC3650755

[B34] Oliveira E. J. D. Aidar S. D. T. Morgante C. V. Chaves A. R. D. M. Cruz J. L. Coelho Filho M. A. (2015). Genetic parameters for drought-tolerance in cassava. *Pesq. Agropec. Bras.* 50 233–241. 10.1590/s0100-204x2015000300007

[B35] Park S. Y. Peterson F. C. Mosquna A. Yao J. Volkman B. F. Cutler S. R. (2015). Agrochemical control of plant water use using engineered abscisic acid receptors. *Nature* 520 545–548. 10.1038/nature14123 25652827

[B36] Patanun O. Ueda M. Itouga M. Kato Y. Utsumi Y. Matsui A. (2017). The histone deacetylase inhibitor suberoylanilide hydroxamic acid alleviates salinity stress in cassava. *Front. Plant Sci.* 7:2039. 10.3389/fpls.2016.02039 28119717 PMC5220070

[B37] Rasheed S. Bashir K. Kim J. M. Ando M. Tanaka M. Seki M. (2018). The modulation of acetic acid pathway genes in *Arabidopsis* improves survival under drought stress. *Sci. Rep.* 8:7831. 10.1038/s41598-018-26103-2 29777132 PMC5959891

[B38] Sako K. Kim J. M. Matsui A. Nakamura K. Tanaka M. Kobayashi M. (2016). Ky-2, a histone deacetylase inhibitor, enhances high-salinity stress tolerance in *Arabidopsis thaliana*. *Plant Cell Physiol.* 57 776–783. 10.1093/pcp/pcv199 26657894

[B39] Savvides A. Ali S. Tester M. Fotopoulos V. (2016). Chemical priming of plants against multiple abiotic stresses: mission possible? *Trends Plant Sci.* 21 329–340. 10.1016/j.tplants.2015.11.003 26704665

[B40] Tang T. Yu A. Li P. Yang H. Liu G. Liu L. (2016). Sequence analysis of the Hsp70 family in moss and evaluation of their functions in abiotic stress responses. *Sci. Rep.* 6:33650. 10.1038/srep33650 27644410 PMC5028893

[B41] Turyagyenda L. F. Kizito E. B. Ferguson M. Baguma Y. Agaba M. Harvey J. J. (2013). Physiological and molecular characterization of drought responses and identification of candidate tolerance genes in cassava. *AoB Plants* 5 lt007. 10.1093/aobpla/plt007 23519782 PMC3604649

[B42] Umezawa T. Nakashima K. Miyakawa T. Kuromori T. Tanokura M. Shinozaki K. (2010). Molecular basis of the core regulatory network in ABA responses: sensing, signaling and transport. *Plant Cell Physiol.* 51 1821–1839. 10.1093/pcp/pcq156 20980270 PMC2978318

[B43] Utsumi Y. Tanaka M. Kurotani A. Yoshida T. Mochida K. Matsui A. (2016). Cassava (Manihot esculenta) transcriptome analysis in response to infection by the fungus colletotrichum gloeosporioides using an oligonucleotide-DNA microarray. *J. Plant Res.* 129 711–726. 10.1007/s10265-016-0828-x 27138000

[B44] Utsumi Y. Tanaka M. Morosawa T. Kurotani A. Yoshida T. Mochida K. (2012). Transcriptome analysis using a high-density oligo microarray under drought stress in various genotypes of cassava, an important tropical crop. *DNA Res.* 19 335–345. 10.1093/dnares/dss016 22619309 PMC3415295

[B45] Utsumi Y. Utsumi C. Tanaka M. Ha V. T. Matsui A. Takahashi S. (2017). Formation of friable embryogenic callus in cassava is enhanced under conditions of reduced nitrate, potassium and phosphate. *PLoS One* 12:e0180736. 10.1371/journal.pone.0180736 28806727 PMC5555663

[B46] Varshney R. K. Tuberosa R. Tardieu F. (2018). Progress in understanding drought tolerance: from alleles to cropping systems. *J. Exp. Bot.* 69 3175–3179. 10.1093/jxb/ery187 29878257 PMC5991209

[B47] Yoon J. Y. Hamayun M. Lee S.-K. Lee I.-J. (2009). Methyl jasmonate alleviated salinity stress in soybean. *J. Crop Sci. Biotechnol.* 12 63–68. 10.1007/s12892-009-0060-5

[B48] Zhao P. Liu P. Shao J. Li C. Wang B. Guo X. (2015). Analysis of different strategies adapted by two cassava cultivars in response to drought stress: ensuring survival or continuing growth. *J. Exp. Bot.* 66 1477–1488. 10.1093/jxb/eru507 25547914 PMC4438449

[B49] Zhong L. Zhou W. Wang H. Ding S. Lu Q. Wen X. (2013). Chloroplast small heat shock protein HSP21 interacts with plastid nucleoid protein pTAC5 and is essential for chloroplast development in *Arabidopsis* under heat stress. *Plant Cell* 25 2925–2943. 10.1105/tpc.113.111229 23922206 PMC3784589

[B50] Zou J. J. Li X. D. Ratnasekera D. Wang C. Liu W. X. Song L. F. (2015). *Arabidopsis* CALCIUM-DEPENDENT PROTEIN KINASE8 and CATALASE3 function in abscisic acid-mediated signaling and h2o2 homeostasis in stomatal guard cells under drought stress. *Plant Cell* 27 1445–1460. 10.1105/tpc.15.00144 25966761 PMC4456645

