# Supplementary figures and images for: Acetic Acid Treatment Enhances Drought Avoidance in Cassava (Manihot esculenta Crantz)
Source: Front Plant Sci. 2019 Apr 24;10:521. doi: 10.3389/fpls.2019.00521 (PMC6492040; doi:10.3389/fpls.2019.00521)

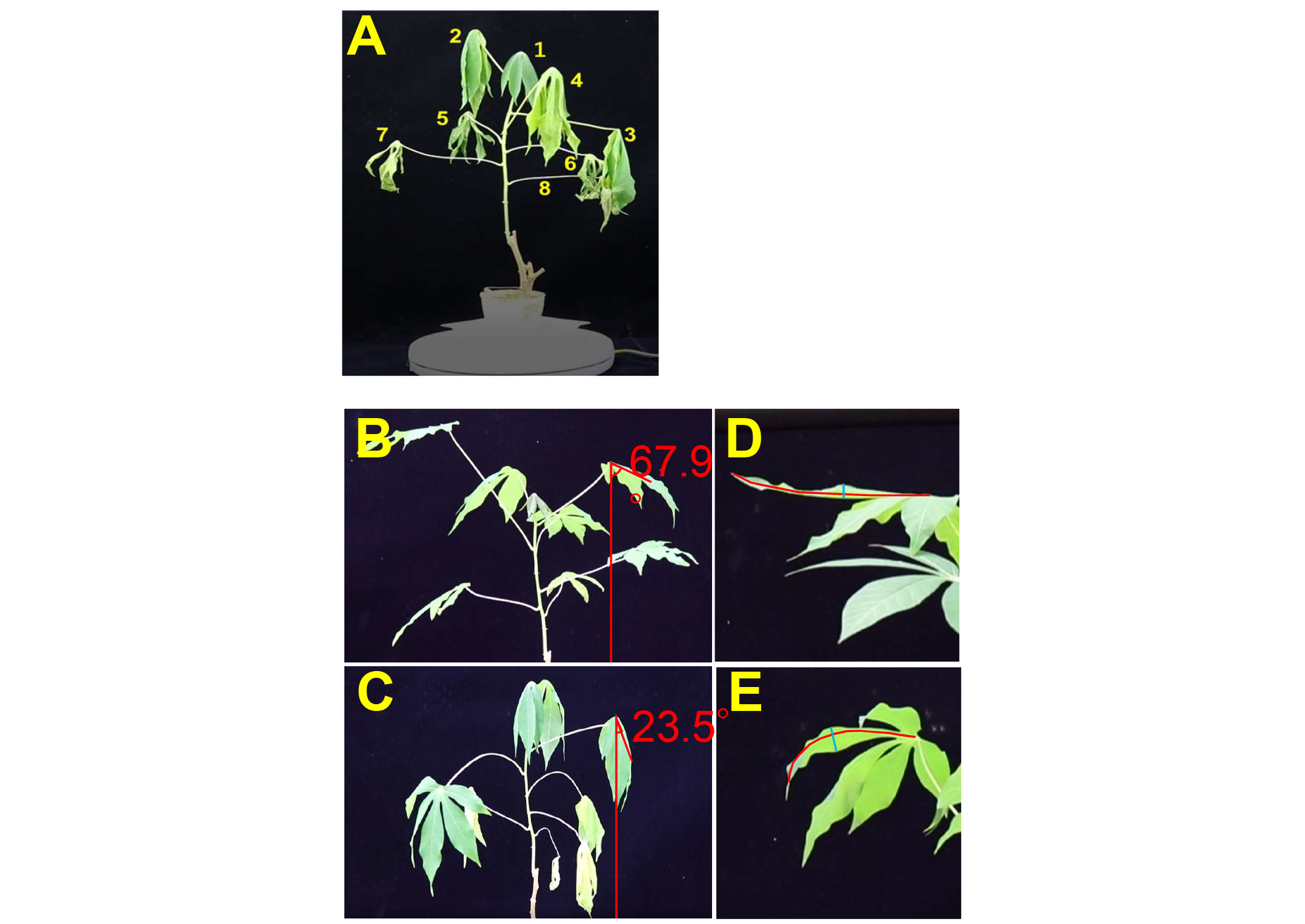

Supplement: Figure S1 — Leaf wilting phenotype of representative water-treated (control) cassava plant after being subjected to a drought (A), representative cassava plant not subjected to a drought (soil drying) (B,D,E), representative cassava plant subjected to a drought stress (C). Images in which the width of the leaflet (blue line) exceeded 10% of the midrib length (red line) were excluded from the analyzed dataset. [file Image_1.TIF]

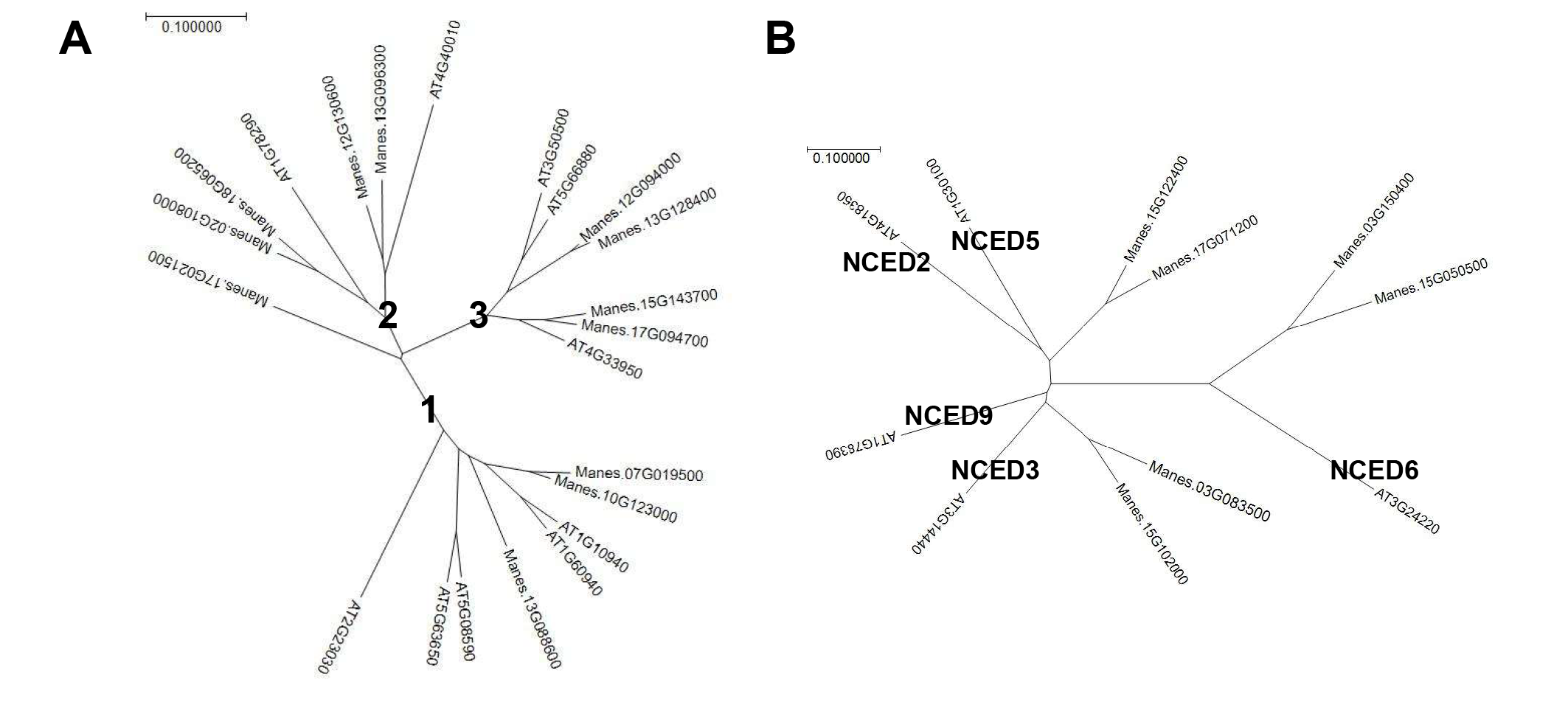

Supplement: Figure S2 — Phylogenetic tree of SnRK2 and NCED amino acid sequences from cassava (Manihot esculenta, Me) and Arabidopsis (Arabidopsis thaliana, At). (A) SnRK2 and (B) NCED amino acid sequences. The distance of the branches denotes the bootstrap majority consensus values on 1,000 replicates. [file Image_2.TIF]
